# Manipulation of Cell Cycle and Chromatin Configuration by Means of Cell-Penetrating Geminin

**DOI:** 10.1371/journal.pone.0155558

**Published:** 2016-05-19

**Authors:** Yoshinori Ohno, Kyoko Suzuki-Takedachi, Shin’ichiro Yasunaga, Toshiaki Kurogi, Mimoko Santo, Yoshikazu Masuhiro, Shigemasa Hanazawa, Motoaki Ohtsubo, Kazuhito Naka, Yoshihiro Takihara

**Affiliations:** 1 Department of Stem Cell Biology, Research Institute for Radiation Biology and Medicine, Hiroshima University, Kasumi, Minami-ku, Hiroshima, Japan; 2 Department of Biochemistry, Faculty of Medicine, Fukuoka University, Nanakuma, Jonan-ku, Fukuoka, Japan; 3 Department of Applied Biological Sciences, College of Bioresource Sciences, Nihon University, Kameino, Fujisawa-city, Kanagawa, Japan; 4 Department of Food and Fermentation Science, Faculty of Food Science and Nutrition, Beppu University, Kita-ishigaki 82, Beppu-city, Oita, Japan; George Mason University, UNITED STATES

## Abstract

Geminin regulates chromatin remodeling and DNA replication licensing which play an important role in regulating cellular proliferation and differentiation. Transcription of the *Geminin* gene is regulated via an E2F-responsive region, while the protein is being closely regulated by the ubiquitin-proteasome system. Our objective was to directly transduce Geminin protein into cells. Recombinant cell-penetrating Geminin (CP-Geminin) was generated by fusing Geminin with a membrane translocating motif from FGF4 and was efficiently incorporated into NIH 3T3 cells and mouse embryonic fibroblasts. The withdrawal study indicated that incorporated CP-Geminin was quickly reduced after removal from medium. We confirmed CP-Geminin was imported into the nucleus after incorporation and also that the incorporated CP-Geminin directly interacted with Cdt1 or Brahma/Brg1 as the same manner as Geminin. We further demonstrated that incorporated CP-Geminin suppressed S-phase progression of the cell cycle and reduced nuclease accessibility in the chromatin, probably through suppression of chromatin remodeling, indicating that CP-Geminin constitutes a novel tool for controlling chromatin configuration and the cell cycle. Since Geminin has been shown to be involved in regulation of stem cells and cancer cells, CP-Geminin is expected to be useful for elucidating the role of Geminin in stem cells and cancer cells, and for manipulating their activity.

## Introduction

Geminin regulates DNA replication licensing through direct interaction with Cdt1, a DNA replication licensing factor, to prevent re-replication, while Geminin may also be involved in regulation of cell cycle progression from the G_0_/G_1_ phase to the S phase, and regulates chromatin remodeling through direct interaction with Brahma/Brg1, a catalytic subunit of the chromatin remodeling factor, SWI/SNF [1,2,3,4,5]. The coiled-coil domain in Geminin is required for the multimerization and the resultant interaction with Cdt1 [6,7,8,9], which possesses a Brahma/Brg1-interacting domain in the C-terminal portion [5,9]. Geminin is further implicated in transcriptional regulation through direct interaction with the Polycomb-group (PcG) complex 1 (also known as the Polycomb repressive complex), a subset of Hox [9,10] and Six3, a homeodomain transcription factor [11]. Geminin protein expression is high in the S/G_2_/M phase, but is down-regulated in the G_0_/G_1_ phase through the ubiquitin-proteasome system including Anaphase Promoting Complex/Cyclosome (APC/C) [1]. The destruction box in Geminin functions as a substrate recognition region for APC/C. As we previously reported, expression of Geminin protein is additionally regulated by the other ubiquitin ligases, including the PcG complex 1 [12] and Cul4a-Ddb1-Roc1 incorporated with Hoxa9/Hoxb4 [13,14], which play a crucial role in sustaining hematopoietic stem cell (HSC) activity [12,13,15,16,17,18]. Geminin expression is thus regulated at the protein level through the ubiquitin-proteasome system containing multiple E3 ubiquitin ligases. Geminin-deficient mice showed that Geminin is essential for development [19,20] as it acts as a central regulator in governing cellular differentiation and proliferation of embryonic stem (ES) and embryonic carcinoma (EC) cells [21] as well as in supporting hematopoietic stem cell (HSC) activity and mature blood cell production [12,13,14,18,22,23]. Expression of Geminin mRNA is high in HSCs and is down-regulated in the progeny subpopulations, which gives credence to the notion that high Geminin expression induces quiescence and an undifferentiated state in HSCs and that the down-regulation provides cellular proliferation capacity and differentiation for the progeny [12]. Thus, constitutive overexpression or constitutive down-regulation of Geminin expression cannot be sufficient for verifying the biological function of Geminin and further for manipulating the cellular function. In this study we generated cell-penetrating Geminin (CP-Geminin) by fusing Geminin with a membrane translocating motif (MTM) of FGF4 and attempted direct transduction of CP-Geminin into cells [24,25]. We also traced the kinetics of incorporation of CP-Geminin into cells and characterized the molecular function as well as the subsequent biological function of incorporated CP-Geminin in NIH 3T3 cells and mouse embryonic fibroblasts (MEF cells). Our findings provide support for the implication that CP-Geminin is a tool for manipulating the cell cycle and chromatin structure.

## Materials and Methods

No human subjects were included in this study, and animal and recombinant DNA experiments were done under the appropriate guidelines approved by Hiroshima University.

### Cell culture

Cells of the mouse fibroblast cell line NIH 3T3 and MEF cells from C57BL/6N embryos (15.5 days post coitus) were grown in Dulbecco’s modified Eagle’s medium (DMEM)(Thermo Fisher Scientific, Waltham, MA) supplemented with 10% fetal bovine serum (FBS) (GE Healthcare UK, Little Chalfont, Buckinghamshire, England), penicillin (100 U/ml) and streptomycin (100 μg/ml) (Wako Pure Chemical, Osaka, Japan) at 37°C in 5% CO_2_. Cells of the mouse leukemic monocyte macrophage cell line Raw 264.7 and of the human chronic myelogenous leukemic cell line K562 were cultured in RPMI 1640 medium (Thermo Fisher Scientific) supplemented with 10% FBS.

### Plasmid construction

Flag-Geminin was generated from a mouse cDNA for *Geminin* by PCR means of PCR amplification using the following primers: sense, 5’-GGATCCGAATTCGACTACAAAGACGATGACGACAAGATGAATCTCAGAATGAAGCAGAAGCAG-3’ and antisense, 5’-GCGGCCGCAAGCTTGTCGACCTGTACACGGCCTAGCATCCGTGGA, and Pfu turbo Hotstart DNA polymerase (Agilent Technologies, Santa Clara, CA). Taq DNA polymerase (Thermo Fisher Scientific) was used to add an A-tail to the PCR product, which was then ligated into the pGEM-T Easy vector (Promega, Madison, WI). The Flag-Geminin vector was digested by using *Not*I and *EcoR*I restriction enzymes, and the resultant fragments were subcloned into the *Not*I and *EcoR*I sites of pET28a or the pET28a-MTM vector containing 6xhis [12]. Flag-, HA-, and 6Myc-tagged cDNAs were subcloned into the down-stream to the CMV promoter of the pcDNA3.1 expression vector (Thermo Fisher Scientific) [26].

### Purification of recombinant proteins

pET28a-Flag-Geminin or pET28a-Flag-Geminin-MTM was transfected into *E*. *coli*, BL21 Star(DE3)pLysS (Thermo Fisher Scientific). The transformants were cultured overnight in LB medium, containing 25 μg/ml kanamycin and 34 μg/ml chloramphenicol (Sigma-Aldrich, St. Louis, MD). Overnight cultures were inoculated into fresh LB medium containing the antibiotics and further cultured for 4 hours (h). Recombinant protein expression was then induced by treatment with isopropyl 1-thio-ß-D-galactopyranoside (IPTG)(Wako Pure Chemical) at a concentration of 0.3 mM. Two h after the induction, cells were collected by centrifugation, and cell pellets were resuspended in suspension buffer [20 mM Tris-HCl (pH7.5), 150 mM NaCl, 0.1% triton X-100 (Wako Pure Chemical), and 0.1 mM phenylmethylsulfonyl fluoride (Nacalai Tesque, Kyoto, Japan)]. The suspensions were sonicated with Digital Ultrasonic Processor Cell Disruptors (Thermo Fisher Scientific) for 2 cycles (turning on and off for 30 seconds during each cycle) and washed in a suspension buffer. The inclusion body was, then, lysed with a guanidine buffer [6 M Guanidine-HCl (Wako Pure Chemical), 457 mM NaCl, 1.2 mM NaH_2_PO_4_, 23.8 mM Na_2_HPO_4_] and the recombinant Geminin proteins were purified by means of histidine-affinity column chromatography with Cobalt-Agarose (Wako Pure Chemical). Next, the purified proteins were refolded through two-step dialysis. First, recombinant proteins were dialyzed against an arginine buffer [0.5 M l-arginine (Wako Pure Chemical), 150 mM Na_4_P_2_O_7_-10H_2_O, 0.01% Tween 80 (Wako Pure Chemical)] overnight at 4°C and then exhaustively dialyzed against PBS. The recombinant protein was separated by SDS-PAGE and was stained by using Coomassie Brilliant Blue (CBB) Stain One Super (Nacalai Tesque) to confirm purification of the recombinant protein [12,13].

### Monitoring of the recombinant proteins

Purified recombinant protein was conjugated with fluorescein isothiocyanate (FITC) by using the Fluorescein Labeling Kit (Dohjindo Laboratories, Mashiki-machi, Kumamoto, Japan) and the intracellular localization was monitored. The nucleus was stained with NucBlue Live Ready Probes reagent (Thermo Fisher Scientific). The cells were incubated with FITC-conjugated proteins or an equimolar concentration of FITC-conjugated BSA and then treated with proteinase K at 0.5 μg/ml for 10 min to remove the non-specifically bound cell-surface proteins. And the cells were examined with a confocal microscope (LSM 5 PASCAL; Carl Zeiss Microscopy, Jena, Germany).

### Immunoprecipitation and immunoblot analysis

Cell were lysed with a lysis buffer consisting of 50 mM Hepes (pH 8.0), 150 mM NaCl, 1.0% Triton X-100 (Wako Pure Chemical), and a protease inhibitor cocktail, the Complete Mini EDTA-free (Roche Diagnostics GmbH, Mannheim, Germany), sonicated for 30 sec on ice and centrifuged for 15 min at 15,000 x g. The supernatant of the lysates was immunoprecipitated with a monoclonal antibody against Flag tag (Medical & Biological Laboratories, Nagoya, Japan), followed by incubation with GammaBind G Sepharose (GE Healthcare). Proteins were separated by means of SDS-PAGE, transferred to Immobilon-P (Merck-Millipore, Billerica, MA), immunoblotted with primary antibodies, and visualized with a horseradish peroxidase-conjugated anti-rabbit IgG antibody and SuperSignal West Femto Maximum Sensitivity substrate (Thermo Fisher Scientific) [26].

### Cell cycle analysis

NIH 3T3 and MEF cells were pulse-labeled with 10 μM 5-Bromo-2'-deoxyuridine (BrdU) for 45 minutes. BrdU incorporated into the nucleus was detected with an APC BrdU-Flow Kit (BD Biosciences, San Diego, CA), and a flow cytometer (FACSCalibur; BD Biosciences) was used for cell sorting analysis.

### Nuclease accessibility analysis

Accessibility of nuclease to the chromatin was examined with the aid of the EpiQ chromatin analysis kit (Bio-Rad, Hercules, CA) in accordance with the manufacturer's protocol with minor modifications [27,28]. Briefly, NIH 3T3 cells were harvested in EpiQ chromatin buffer, then treated with EpiQ nuclease for 1h at 37°C followed by DNA extraction. Nuclease sensitivity for the E2F-responsive region (E2F-R) [29] was assessed by PCR analysis with the primer sets described previously [26]. Nuclease accessibility is defined as the susceptibility of the targeted genomic DNA region to nuclease digestion. The index of the target genomic DNA region after nuclease treatment is calculated relative to that of the reference gene region of the *ß-globin* gene, which is not susceptible to nuclease digestion in NIH 3T3 cells. The EpiQ Chromatin Kit Data Analysis Tool (www.bio-rad.com/epiq) was used for analysis of the data [26].

### Cell transfection

Plasmid DNAs were transfected into NIH 3T3 cells by using lipofectamine 2000 (Life Technologies). Six h after the transfection, cells were washed, and the medium was replaced with a fresh one, after which the transfectants were subjected to further analyses [26].

### Luciferase assay

The cells were lysed 24h after transfection, and the luciferase assay was performed by using the Dual-Glo luciferase assay system in accordance with the manufacturer’s protocol (Promega, Madison, WI). We used the pGL3-basic firefly luciferase reporter vector encompassed within the promoter region of the human *Geminin* genes [29] and a Renilla luciferase reporter plasmid, pE2MTx4-Renilla [30]. To normalize transfection efficiency, firefly luciferase values were standardized to the values for Renilla luciferase [26].

### TaqMan real-time PCR

Total RNA was purified from cells with the Quick-RNA MicroPrep Kit (ZYMO Research, Irvine, CA), and was reverse-transcribed with the aid of TaqMan Reverse Transcription Reagents (Thermo Fisher Scientific). The resultant cDNA was then subjected to quantitative real-time PCR analysis by using TaqMan Gene Expression Assays and an Applied Biosystems 7500 real-time PCR system (Thermo Fisher Scientific). The relative expression level of the mRNA was determined by normalization to the mRNA level of glyceraldehyde 3-phosphate dehydrogenase (GAPDH) [26].

### Antibodies

Primary and secondary antibodies used are listed in S1 Table.

### Statistical analysis

More than three independent experiments were done, and the data were analyzed with the Student’s t-test. The results are shown with standard errors of measurement (SEMs).

## Results

### Generation of CP-Geminin protein

**Fig 1:**
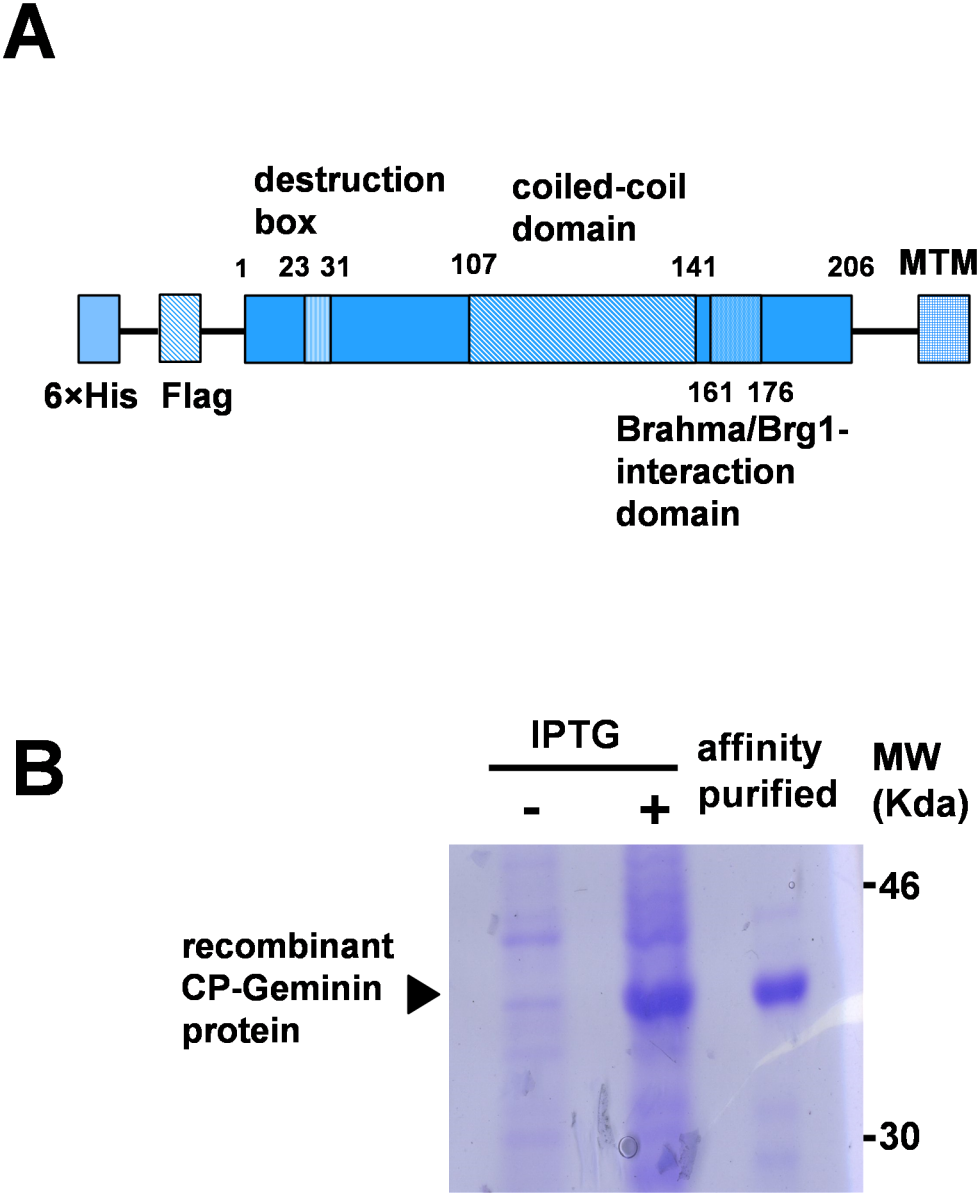
Generation of recombinant CP-Geminin protein (A) Structure of CP-Geminin. Amino acid numbers indicate location of the representative domains. The coiled-coil domain is required for the dimerization or multimerization and also for the interaction with Cdt1. MTM: membrane translocating motif from FGF4. (B) Production and affinity-purification of recombinant CP-Geminin. Proteins were separated on SDS-PAGE and subjected to CBB staining. MW: molecular weight.

Our aim was to manipulate an expression level of Geminin by directly transducing Geminin protein into cells. For this purpose, we generated a CP-Geminin recombinant protein. A cDNA for mouse Geminin was inserted into the pET28a-Flag plasmid vector with or without MTM from FGF4 (Fig 1A). The protein was expressed in *E*. *coli*, and was affinity-purified under denaturing conditions. The recombinant protein was separated by SDS-PAGE, and was confirmed by means of CBB staining (Fig 1B).

### Transduction of a CP form of Geminin protein into cells

**Fig 2:**
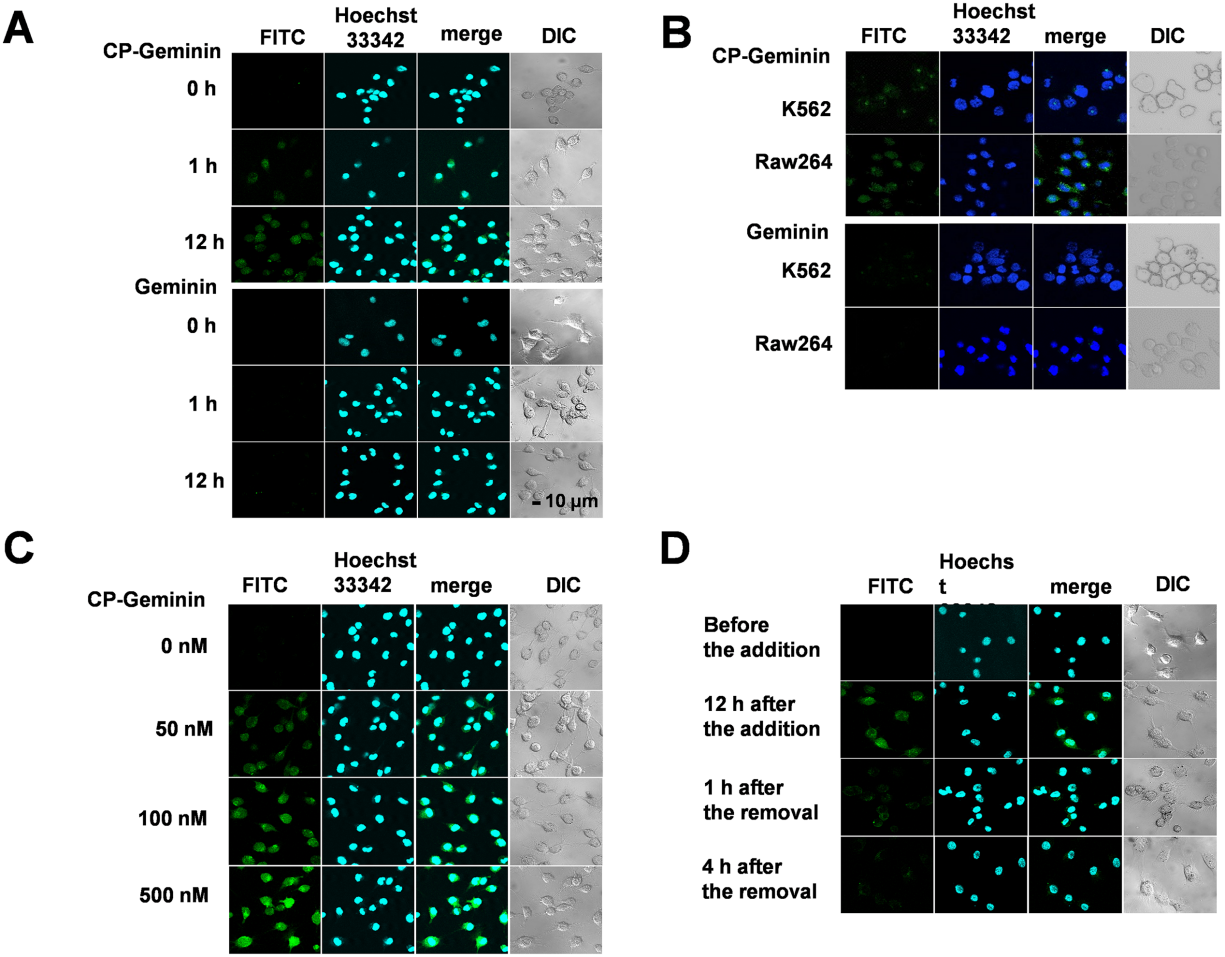
Geminin transduction by means of CP-Geminin (A) CP-Geminin incorporation into NIH 3T3 cells. FITC-conjugated CP-Geminin and Geminin were added into the medium. One and 12h after the addition, cells were observed under a confocal microscope. The nucleus was stained with Hoechst33342. DIC: differential interference contrast (B) FITC-conjugated CP-Geminin incorporation into leukemic cell lines 24 h after the addition. (C) Dose dependency of CP-Geminin incorporation into NIH 3T3 cells. FITC-conjugated CP-Geminin was added to the medium at the indicated concentration, and 12h after the addition, the cells were examined under a confocal microscope. (D) Withdrawal study of CP-Geminin. FITC-conjugated CP-Geminin was added to the medium and the incorporation was confirmed 12h after the addition. CP-Geminin was then removed by replacing the medium with CP-Geminin with one without CP-Geminin. One and 4h after the removal, the cells were examined under a confocal microscope.

We conjugated CP-Geminin recombinant protein with FITC to trace its incorporation into cells. Intracellular incorporation of FITC-conjugated CP-Geminin was examined under a confocal microscope, showing that CP-Geminin was rapidly incorporated into NIH 3T3 cells, while recombinant Geminin was not (Fig 2A). We further examined whether FITC-conjugated CP-Geminin was also incorporated into K562 and Raw264.7 cells and confirmed that CP-Geminin was incorporated into these cell lines but that recombinant Geminin was not (Fig 2B), although the incorporation was less efficient in these cell lines than in NIH 3T3 cells (Fig 2B). Next we examined the kinetics of CP-Geminin incorporation into NIH 3T3 cells (Fig 2A), which was detected as little as 1h after the addition to the medium, and the incorporated CP-Geminin increased in a time-dependent manner until 12h after the addition. The incorporation was observed in the nucleus as well as in the cytoplasm. Twelve h after addition to the medium, there was more incorporated CP-Geminin in the nucleus than in the cytoplasm. We also examined dose-dependency of the incorporation. After the addition of CP-Geminin at 50 nM, 100 nM or 500 nM, respectively, the incorporated CP-Geminin increased depending on the dose of CP-Geminin added to the medium (Fig 2C). We next conducted a withdrawal study to trace the dynamics of the incorporated CP-Geminin. After the addition of CP-Geminin at 50 nM, incorporation into the cells was confirmed 12h after the addition. We then removed CP-Geminin by placing the medium with CP-Geminin by one without CP-Geminin, after which the incorporated CP-Geminin rapidly decreased and little CP-Geminin remained detectable as little as 1h after the removal (Fig 2D), indicating the half-life of incorporated CP-Geminin is less than 1h. We added FITC-conjugated CP-Geminin at 1,000 nM into culture medium of culture primary cells, MEF cells. Incorporated FITC-conjugated CP-Geminin was found in the nucleus as well as in the cytoplasm (S1 Fig). The incorporation was, however, less efficient in MEF than in NIH 3T3 cells.

### Molecular function of CP-Geminin

**Fig 3:**
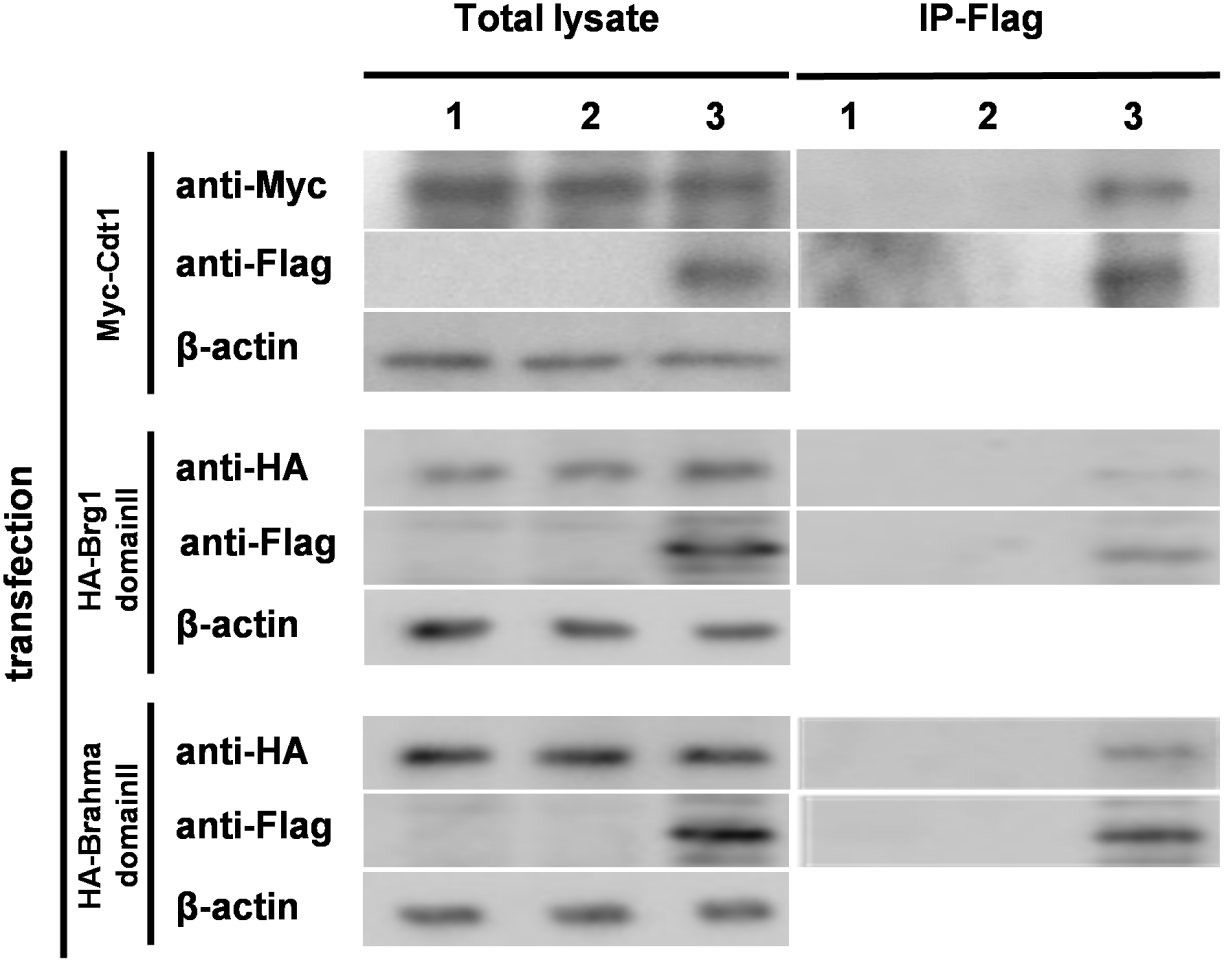
Molecular interaction of incorporated CP-Geminin with the target substrates. Expression plasmid vectors for Myc-Cdt1, HA-Brahma-domainII and HA-Brg1-domainII were transfected into NIH 3T3 cells, after which CP-Geminin was added. The cells were then subjected to immunoprecipitation analysis. 1. Control, 2. Flag-Geminin, 3. CP-Geminin including a Flag tag.

It has been shown by the immunoprecipitation analysis that Geminin directly interacts with Cdt1 and Brahma/Brg1. For the current study we biochemically examined whether cell-incorporated CP-Geminin directly interacts with Cdt1 and Brahma/Brg1-domainII in a manner similar to that observed with endogenous Geminin [5,26]. We transduced CP-Geminin into NIH 3T3 cells and further transfected each of the expression vectors for Cdt1 and Brahma/Brg1-domainII, and 48h after the transfection, the incorporated CP-Geminin was subjected to immunoprecipitation analysis. Since Flag-tag was incorporated for CP-Geminin, Myc-tag for Cdt1, and HA-tag for Brahma/Brg1-domainII, we could immunoprecipitate CP-Geminin by using an anti-Flag antibody and the immunoprecipitants were then subjected to immunoblot analysis. Cdt1 and Brahma/Brg1-domainII were unmistakably detected when each of the antibodies against the corresponding tags was used (Fig 3), indicating that cell-incorporated CP-Geminin directly interacted with Cdt1 and Brahma/Brg1 in a manner similar to that of each of the endogenous molecules [2,3,5].

**Fig 4:**
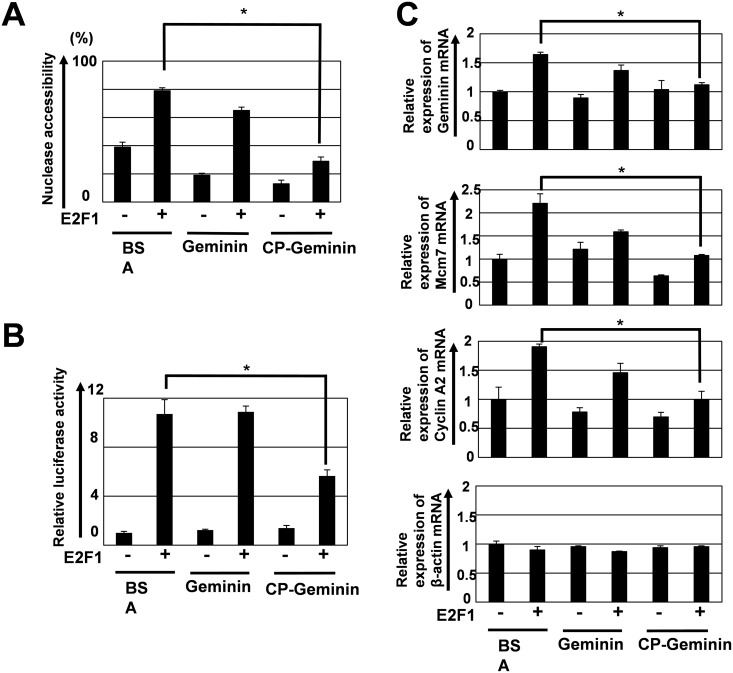
Effect of CP-Geminin on chromatin configuration and transcription. (A) Effect of CP-Geminin on nuclease accessibility of the E2F-R chromatin locus of the *Geminin* gene. Twenty-four h after transduction of CP-Geminin (1,000 nM), cells were subjected to nuclease accessibility analysis. BSA, control (B) Effect on transcription of the *Geminin* gene promoter. Twenty four h after transduction of CP-Geminin (1,000 nM), E2F1 (1 μg) and the luciferase reporter plasmids (1.5 μg) were co-transfected, and transcription activity of the *Geminin* gene promoter was examined with the luciferase reporter assay. (C) Effect of CP-Geminin on mRNA expression of the endogenous genes, *Geminin*, *Mcm7*, *CcnA2* and *Actb2*. Twenty four h after transduction of CP-Geminin (1,000 nM), E2F1 (1 μg) was transfected, and cells were subjected to TaqMan real-time PCR analysis an additional 24h after the transfection. *: P< 0.01.

Next, we examined whether cell-incorporated CP-Geminin could generate change in the chromatin structure. We previously reported that plasmid-mediated overexpression of Geminin altered nuclease accessibility in E2F-R located in the first intron of the *Geminin* gene to repress transcription, suggesting that overexpressed Geminin altered the chromatin configuration to negatively regulate Geminin transcription, *i*.*e*., Geminin regulates transcription of the *Geminin* gene itself through a negative feed-back mechanism [26]. For the current study we examined whether cell-incorporated CP-Geminin changes chromatin configuration in a similar manner. We focused on E2F-R in the *Geminin* gene [26,29], and examined the effect of CP-Geminin on accessibility of EpiQ nuclease to the E2F-R chromatin locus. As was shown previously, exogenous transfected E2F1 induces nuclease accessibility to the E2F-R chromatin locus [26]. We found that CP-Geminin suppressed the E2F1-mediated augmented nuclease accessibility (Fig 4A), while recombinant Geminin did so less efficiently.

We next examined the effect of CP-Geminin on E2F-mediated transcriptional activation by using a luciferase reporter for the *Geminin* gene promoter which contained E2F-R. The transient transfection experiments showed that CP-Geminin suppressed E2F1-induced transcriptional activation of the reporter gene (Fig 4B). We further examined the effect of CP-Geminin on E2F-mediated augmentation of mRNA expression of the *Geminin*, *Mcm7* and *Cyclin A2* (*Ccna2*) genes (Fig 4C). These genes are known to be regulated by members of the E2F family [29,31,32]. Although expression of mRNA for the *ß-actin* (*Actb*) gene, an E2F-unresponsive control gene, was not affected by either transfection of E2F1 or by the addition of recombinant Geminin and CP-Geminin (Fig 4C), addition of CP-Geminin suppressed E2F-1-mediated augmentation of mRNA expression of the *Geminin* gene, while that of recombinant Geminin did so less efficiently (Fig 4C). These led us to presume that the effect of CP-Geminin on chromatin configuration affected E2F1-mediated transcriptional activation in the *Geminin* gene. And a similar transcriptional suppressive effect of CP-Geminin on the *Mcm7* and *Cyclin A2* (*Ccna2*) genes (Fig 4C) was observed, suggesting that the effect of CP-Geminin on chromatin configuration is not limited to E2F-R in the *Geminin* gene but extends to the genomic loci of the other E2F-responsive genes. These findings are highly compatible with the assumption that CP-Geminin suppresses chromatin remodeling through direct interaction with Brahma/Brg1 to repress the transcription, because we previously showed that plasmid transfection-mediated Geminin overexpression reduced nuclease accessibility and transcription and that Geminin5EQ, lacking an interaction capacity with Brahma/Brg1, exerted a dominant-negative effect on endogenous Geminin to induce nuclease accessibility and the transcription [26]. The chromatin loci being regulated by the SWI/SNF chromatin remodeling complexes could thus be candidate target loci for CP-Geminin to alter chromatin configuration and transcription.

### Biological function of CP-Geminin

**Fig 5:**
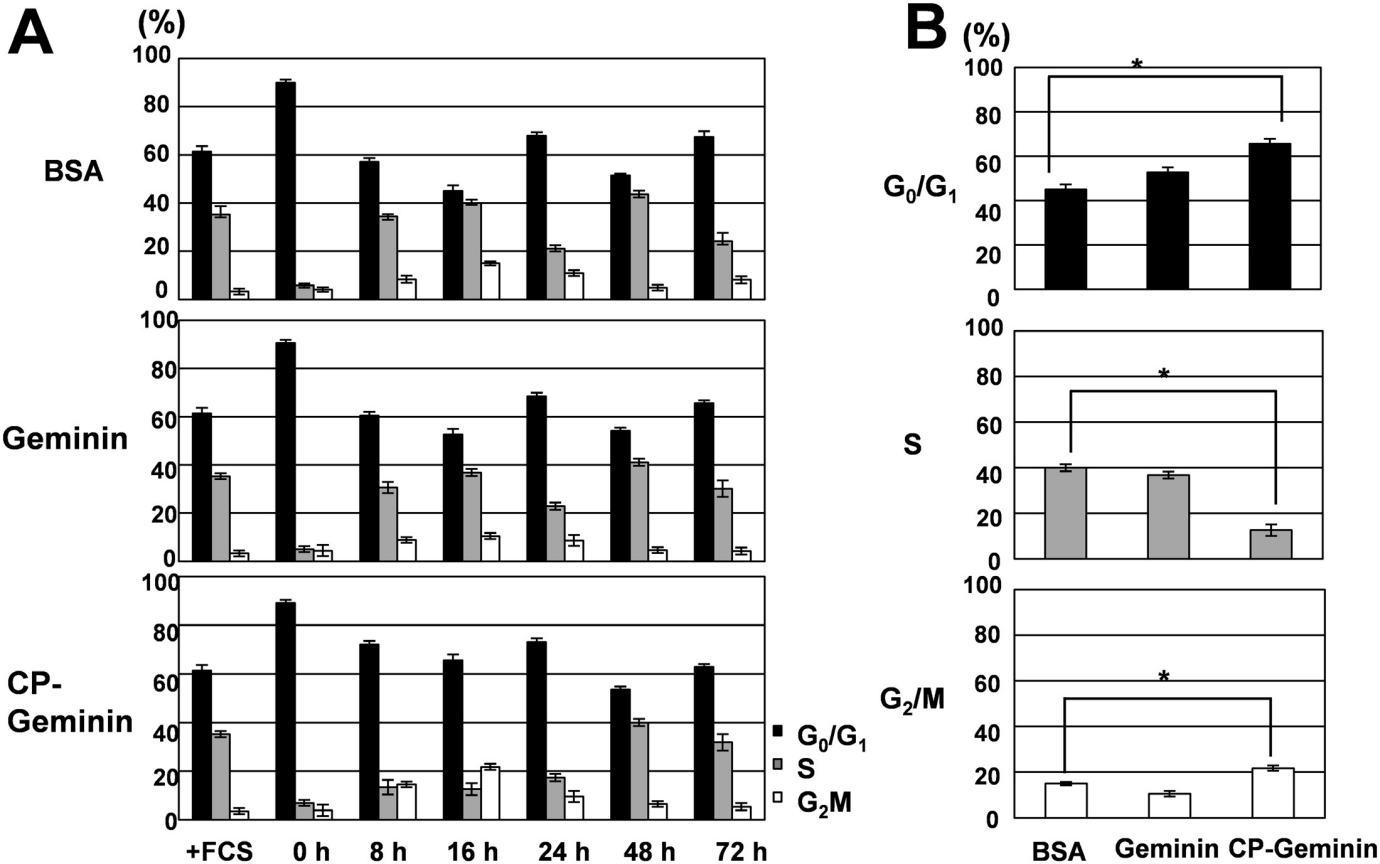
Biological function of CP-Geminin. (A) CP-Geminin was transduced into synchronized NIH 3T3 cells by means of serum depletion, and its effect on the cell cycle was observed until 72 h after serum induction. (B) Cell cycle status 16h after serum induction. S-phase progression was markedly suppressed by CP-Geminin. *: P< 0.01.

Geminin has been shown to suppress DNA replication licensing through the direct interaction with Cdt1, which prevents DNA re-replication in the S phase of the cell cycle, and Geminin is also presumed to be involved in the cell cycle progression. In our study, we arrested the cell cycle by means of serum depletion in NIH 3T3 cells for 48 h and induced the cell cycle by addition of serum at 20%. We then examined the effect of CP-Geminin at 500 nM on the cell-cycle progression. S-phase progression was induced by serum addition and the percentage of S-phase cells reached approximately 40% in cells cultured with BSA or recombinant Geminin 16h after serum induction, while as little as 10% was detected in those cultured with CP-Geminin (Fig 5A and 5B). CP-Geminin thus suppressed S-phase progression of the cell cycle, while recombinant Geminin and BSA did not. Similar suppression for S-phase progression by retrovirus-mediated Geminin transduction was observed (S2 Fig). Then we examined effect of CP-Geminin on the cell cycle in MEF cells. Twelve hours after the serum induction, S-phase progression was significantly suppressed by the addition of CP-Geminin, which was statistically significant, but less efficient than in NIH 3T3 cells (S3 Fig). As we mentioned above, incorporated CP-Geminin directly interacted with Cdt1 and also with Brahma/Brg1, leading us to presume that incorporation of CP-Geminin suppressed the cell cycle progression through the direct interaction with Cdt1 and Brahma/Brg1.

## Discussion

Direct protein transduction has been achieved by using the hydrophobic region of a signal peptide sequence [24,33], MTM from FGF4 [25,34,35], TAT from human immunodeficiency virus [4,36], VP22 from herpes simplex virus [37], Antp [24], HHph-1 [38], R7 [39] and R9 [40]. For our study we generated a bacterial recombinant CP-Geminin protein into which MTM from FGF4 was incorporated. Geminin is reportedly a nuclear protein and mainly exerts its molecular functions in the nucleus [1,41,42]. Involvement of Cdt1 in the nuclear transfer has been pointed out, and nuclear-to-cytoplasm shuttling was also reported in the subcellular localization of Geminin [43,44]. We were able to show that CP-Geminin was incorporated first into the cytoplasm, and subsequently imported into the nucleus. Incorporated CP-Geminin is thus presumed to exert its molecular functions mainly in the nucleus. Since the Geminin protein is regulated by the ubiquitin-proteasome system with APC/C [1], PcG complex 1 [12] and Cul4a-Ddb1-Roc1 incorporated with Hoxa9/b4 [13,14], the stability of CP-Geminin may also be regulated by these systems. The stability of incorporated CP-Geminin may thus vary among cells and may also depend on the cell-cycle phase in which cells reside.

Geminin inhibits the molecular functioning of Cdt1 [2,3] as well as Brahma/Brg1 [5]. Cdt1 is required for loading of the prereplicative complex, while SWI/SNF chromatin remodeling complexes, including Brahma/Brg1, are involved in E2F-Rb-mediated transcriptional regulation of the genes for cell cycle and DNA replication machineries [45]. These functions may be molecular events underlying CP-Geminin-mediated suppression of the S-phase progression in NIH 3T3 cells. Since it has been put forward that DNA replication licensing, *i*.*e*., chromatin loading of the prereplicative complex, is a molecular event defining the G_1_ phase in the cell cycle [42], Geminin-mediated S-phase suppression may be based on suppression of G_1_-phase progression from the G_0_ phase, which suggests that CP-Geminin is useful for inducing cells into the G_0_ phase. Geminin, on the other hand, stabilizes the Cdt1 protein, which may help secure DNA replication licensing in the next round of the cell cycle [46,47]. This finding seems to implicate that Geminin plays an important biological role in providing cells, such as stem cells, with proliferation potential. Curiously, Geminin mRNA expression is higher in the long-term repopulating HSC subpopulation than in their progeny subpopulations [12]. These findings suggest the possibility that Geminin acts as a regulator for cellular proliferation and differentiation in the stem cells, *i*.*e*., high Geminin mRNA expression may help maintain cellular quiescence and undifferentiated states in the stem cells, while down-regulation of Geminin may induce cellular differentiation and proliferation to produce mature blood cells. A similar role has also been suggested for Geminin in ES and EC cells [21]. Dynamic Geminin expression may thus play an important role in regulating cellular proliferation and differentiation in the stem cell system. A new technology for manipulating expression levels of Geminin during a specific period and also at a specific level, *i*.*e*., CP-Geminin, could help further elucidate the role of Geminin in stem cells and cancer cells, and could also provide a new tool for controlling their activity.

## Supporting Information

S1 Fig CP-Geminin incorporation into MEF cells. FITC-conjugated CP-Geminin and Geminin (1,000 nM) were added into the medium. Twenty four h after the addition, cells were observed under a confocal microscope. The nucleus was stained with Hoechst33342. DIC: differential interference contrast.
(DOCX)

S2 Fig Effect of retrovirus-mediated Geminin transduction on the cell cycle of NIH 3T3 cells. Geminin was transduced into NIH 3T3 cells with a retrovirus-mediated gene transfer method. We used a mouse stem cell virus vector with an enhanced yellow fluorescence protein gene driven by a phosphoglycerate kinase promoter, labeled as MEP (12). The cells were synchronized at the G_0_ phase by means of serum depletion and the cell cycle status was monitored after serum induction. MEP: an empty vector (A) Cell cycle profiles after serum induction. (B) The cell cycle status 8 h after serum induction. *: P< 0.01.
(DOCX)

S3 Fig Effect of CP-Geminin on the cell cycle of MEF cells. CP-Geminin was transduced into synchronized MEF cells by means of serum depletion, and its effect on the cell cycle was observed until 72 h after serum induction. (B) Cell cycle status 12 h after serum induction. S-phase progression was suppressed by CP-Geminin, which was statistically significant. *: P< 0.01.
(DOCX)

S1 Table Antibodies used in the study. (DOCX)
