# Supplementary material for: Manipulation of Cell Cycle and Chromatin Configuration by Means of Cell-Penetrating Geminin
Source: PLoS One. 2016 May 19;11(5):e0155558. doi: 10.1371/journal.pone.0155558 (PMC4873132; doi:10.1371/journal.pone.0155558)
Supplement: S1 Fig — FITC-conjugated CP-Geminin and Geminin (1,000 nM) were added into the medium. Twenty four h after the addition, cells were observed under a confocal microscope. The nucleus was stained with Hoechst33342. DIC: differential interference contrast. (DOCX) [file pone.0155558.s001.docx]

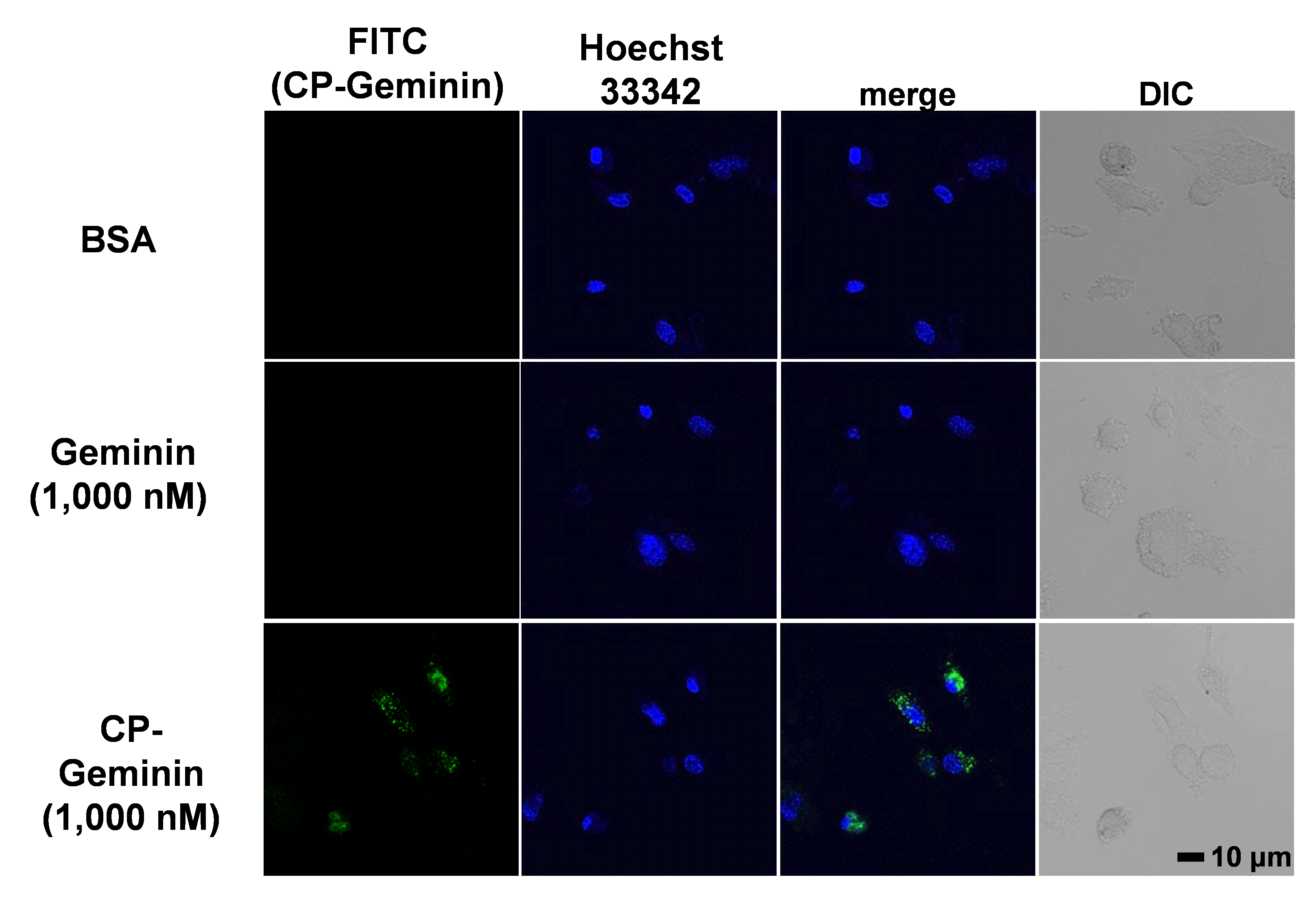


**S1 Fig. CP-Geminin incorporation into MEF cells.** FITC-conjugated CP-Geminin and Geminin (1,000 nM) were added into the medium. Twenty four h after the addition, cells were observed under a confocal microscope. The nucleus was stained with Hoechst33342. DIC: differential interference contrast
