# Supplementary material for: Manipulation of Cell Cycle and Chromatin Configuration by Means of Cell-Penetrating Geminin
Source: PLoS One. 2016 May 19;11(5):e0155558. doi: 10.1371/journal.pone.0155558 (PMC4873132; doi:10.1371/journal.pone.0155558)
Supplement: S2 Fig — Geminin was transduced into NIH 3T3 cells with a retrovirus-mediated gene transfer method. We used a mouse stem cell virus vector with an enhanced yellow fluorescence protein gene driven by a phosphoglycerate kinase promoter, labeled as MEP (12). The cells were synchronized at the G0 phase by means of serum depletion and the cell cycle status was monitored after serum induction. MEP: an empty vector (A) Cell cycle profiles after serum induction. (B) The cell cycle status 8 h after serum induction. *: P< 0.01. (DOCX) [file pone.0155558.s002.docx]

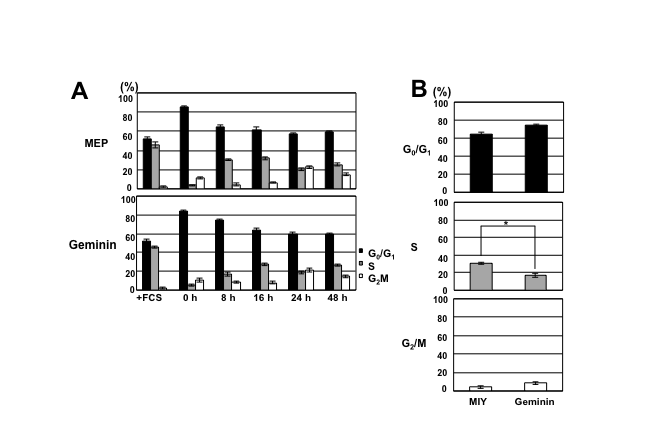


**S2 Fig. Effect of retrovirus-mediated Geminin transduction on the cell cycle of NIH 3T3 cells.** Geminin was transduced into NIH 3T3 cells with a retrovirus-mediated gene transfer method. We used a mouse stem cell virus vector with an enhanced yellow fluorescence protein gene driven by a phosphoglycerate kinase promoter, labeled as MEP ([12](#_ENREF_12)). The cells were synchronized at the G_0_ phase by means of serum depletion and the cell cycle status was monitored after serum induction. MEP: an empty vector (A) Cell cycle profiles after serum induction. (B) The cell cycle status 8 h after serum induction. *: P< 0.01
