# Supplementary material for: Manipulation of Cell Cycle and Chromatin Configuration by Means of Cell-Penetrating Geminin
Source: PLoS One. 2016 May 19;11(5):e0155558. doi: 10.1371/journal.pone.0155558 (PMC4873132; doi:10.1371/journal.pone.0155558)
Supplement: S3 Fig — CP-Geminin was transduced into synchronized MEF cells by means of serum depletion, and its effect on the cell cycle was observed until 72 h after serum induction. (B) Cell cycle status 12 h after serum induction. S-phase progression was suppressed by CP-Geminin, which was statistically significant. *: P< 0.01. (DOCX) [file pone.0155558.s003.docx]

**
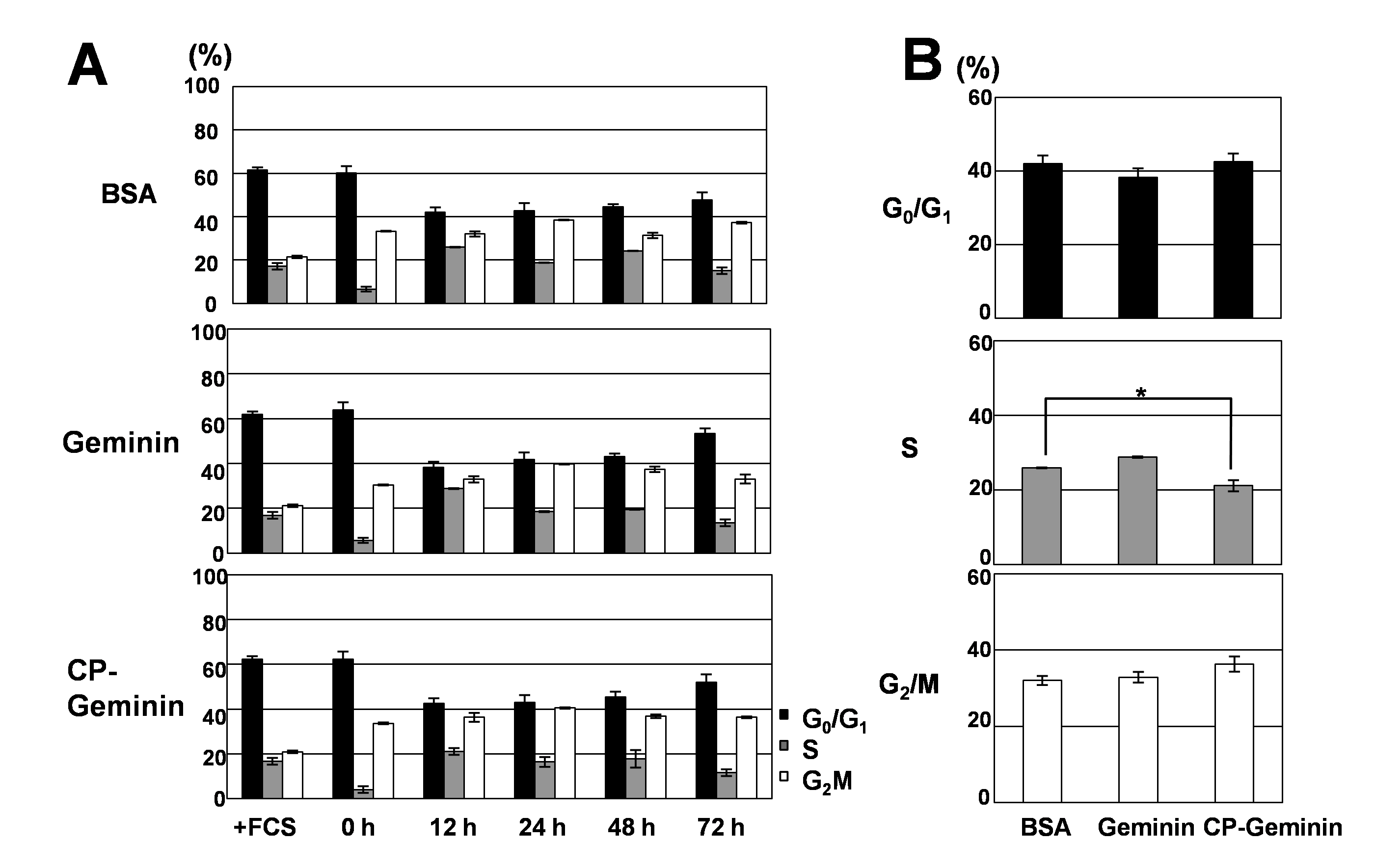
**

**S3 Fig. Effect of CP-Geminin on the cell cycle of MEF cells.**

(A) CP-Geminin was transduced into synchronized MEF cells by means of serum depletion, and its effect on the cell cycle was observed until 72 h after serum induction. (B) Cell cycle status 12 h after serum induction. S-phase progression was suppressed by CP-Geminin, which was statistically significant. *: P< 0.01
