# Supplementary material for: Manipulation of Cell Cycle and Chromatin Configuration by Means of Cell-Penetrating Geminin
Source: PLoS One. 2016 May 19;11(5):e0155558. doi: 10.1371/journal.pone.0155558 (PMC4873132; doi:10.1371/journal.pone.0155558)
Supplement: S1 Table — (DOCX) [file pone.0155558.s004.docx]

**S1 Table. Antibodies used in the study.**

**Antibody** (Clone or Product number) **Species(*) Manufacturer**

**Primary antibody**

anti-DDDDK-tag(anti-Flag-Tag, FLA-1) mouse (m) MBL

anti-DDDDK-tag(anti-Flag-Tag, PM020) rabbit (p) MBL

anti-HA(ab9110) rabbit (p) Abcam

anti-Geminin rabbit (p) #

anti-Myc(sc-789) rabbit (p) Santa Cruz

anti-β-actin(AC-74) mouse (m) Sigma

**Secondary antibody**

anti-rabbit IgG（H+L）(31460) goat (p) Thermo

anti-mouse IgG（H+L）(31430) goat (p) Thermo

* m, monoclonal antibody; p, polyclonal antibody

# Polyclonal antibody raised against the GST-fusion recombinant molecules.

MBL, Medical ＆ Biological Laboratories, Nagoya, Japan; Abcam, Cambridge, UK; Santa Cruz, Santa Cruz Biotechnology, Dallas, TX; Sigma, Sigma-Aldrich, St. Louis, MO; Thermo, Thermo Fisher Scientific, Waltham, MA.
